# Improving the Nutritional Profile of Intermediate Wheatgrass by Solid-State Fermentation with *Aspergillus oryzae* Strains

**DOI:** 10.3390/foods14030395

**Published:** 2025-01-25

**Authors:** Takehiro Murai, George A. Annor

**Affiliations:** Department of Food Science and Nutrition, College of Food, Agricultural and Natural Resource Sciences, University of Minnesota, 1334 Eckles Avenue, St. Paul, MN 55108, USA; murai021@umn.edu

**Keywords:** *Aspergillus oryzae*, fermentation, intermediate wheatgrass

## Abstract

*Aspergillus oryzae* has been used to ferment various cereal grains throughout history, as seen in the examples of sake, soy sauce, and miso. It is known that this fermentation enhances the nutritional quality of the raw materials by breaking down complex molecules into simpler, more digestible forms and increasing the bioactive compounds. In this study, intermediate wheatgrass (IWG) was fermented with three different strains of *A. oryzae* suitable for making sake, soy sauce, and miso. Whole IWG flour was mixed with water (1:2 *w*/*w*), autoclaved at 121 °C for 20 min, cooled, mixed with *A. oryzae* spores, and fermented for seven days at 30 °C. Sugars, protein, amino acids, kojic acid, total phenolic content, total flavonoid content, and DPPH radical scavenging activity were measured. The protein content increased significantly (*p* < 0.05) from 18.0 g/100 g to over 30 g/100 g after seven days. Lysine showed a positive correlation with protein content across all three strains, with its ratio increasing as the protein content increased, while all other essential amino acids displayed a negative correlation and a decreasing ratio with the protein content. Autoclaving increased the verbascose content of IWG, and further increases were observed during the first two days of fermentation across all three strains, followed by a subsequent decline. Peak glucose content was observed on days 3~4 but also decreased in the subsequent days. Total phenolic content, total flavonoid content, kojic acid, and DPPH scavenging activity peaked around day 4~5 for all three strains, followed by a slight decrease in the subsequent days. The findings of this study highlight the potential of solid-state fermentation to improve the nutritional profile of IWG, emphasizing that the selection of *A. oryzae* strains and the fermentation duration can affect the fermentation outcome and nutritional enhancements.

## 1. Introduction

Intermediate wheatgrass (*Thinopyrum intermedium*, IWG) is a perennial cereal grain introduced to the U.S. in the 1930s from Eurasia and was used as forage crops until its domestication in the 1980s [1]. IWG is known for its environmental benefits, such as carbon sequestration and prevention of nitrate leaching, compared to wheat [2]. In addition to the environmental benefits, research by Tyl and Ismail (2019) has shown the superior nutritional benefits, such as higher dietary fiber, protein, and antioxidants, in IWG compared to wheat [3]. However, as IWG is still a relatively new cereal grain in the market with its first food-grade variety, “MN-Clearwater”, released in 2019 [1], its application in the food industry is still under development. In this vein, studies on the functionality of IWG and the effects of processing methods on its functionality have been gaining interest in recent years. Ty et al. (2019) developed tempering conditions for producing refined IWG flour, applicable to wheat-based products like pasta and bread [4]. Research by Cetiner et al. (2023) has shown the controlled addition of IWG flour to improve baking properties such as bread volume and softness [5]. Additionally, Boakye et al. (2022) investigated the extrusion conditions for expanded IWG products [6], while Marcus and Fox (2022) examined the prospects of malting IWG and producing wort [7]. However, no research has been conducted on the effects of fermentation on the nutritional properties of IWG.

Solid-state fermentation (SSF) of cereal grains with fungi has been a common practice throughout human history, as seen in examples such as sake, miso, and soy sauce [8]. Taking the example of koji, fermented for sake production, solid-state fermentation takes place by inoculating steamed rice with koji spores (*A. oryzae* spores) and fermenting it at around 30 °C and between 50 and 80% relative humidity [9]. SSF by various *Aspergillus* strains has been found to produce various hydrolytic enzymes through the fermentation process, improving the bioavailability of nutrients in the cereal grains [10,11]. Research by Zwinkles et al. (2023) has shown that SSF alters the amino acid profile and increases the PDCAAS values of rice and barley [11]. Such ability of *A. oryzae* to improve the protein quality of cereal grains has a potential application in improving animal feed as well [12]. In other applications, the release of sugar from starch content with amylase-type enzymes can be applied to produce a sweet dessert drink, known as amazake, or used as an ingredient in the production of sake [13,14].

The application of SSF goes beyond food processing and into the production of various chemical substances, including bioactive compounds for pharmaceuticals, organic acids for cosmetics, and enzymes for industrial applications [15,16,17]. Given the versatility of SSF and the established benefits of IWG, investigating the effects of SSF on IWG could unlock new opportunities to enhance its nutritional profile and develop innovative food products with ecological advantages over conventional grain-based alternatives. In this study, we ferment IWG with three different strains of *A. oryzae* over 7 days and compare the changes in the nutritional profile to understand the impact of SSF on IWG.

## 2. Materials and Methods

### 2.1. Materials

*A. oryzae* NJK401 (sake koji), *A. oryzae* NJK701 (soy sauce koji), and *A. oryzae* NJK806 (miso koji) were generously provided from Nihon Jouzou Kougyou (Tokyo, Japan). Whole intermediate wheatgrass was obtained from Perrenial Pantry (Burnsville, MN, USA).

### 2.2. Fermentation and Extraction

Whole intermediate wheatgrass was milled to less than 0.5 mm in particle size and mixed with water at a ratio of 1:2 (*w*/*w*). The mixture was then autoclaved at 121 °C for 20 min and cooled to below 30 °C. The sample mixtures were inoculated with *A. oryzae* spores (1% *w*/*w*) and fermented at 30 °C at a humidity of 65% under a draft for 7 days. The fermentation temperature and humidity were based on the standard practices of the koji-making process in sake production [9]. Previous research indicates that the optimal temperature for amylase-type enzyme production during koji fermentation ranges from 35 °C to 40 °C, whereas protease-type enzymes are more abundantly produced at 30 °C [18]. For this experiment, a temperature of 30 °C was selected to account for the higher protein content of IWG compared to rice for a more efficient fermentation [3,19]. The samples for analysis were collected from four different sections of the mixture every 24 h, sterilized in an autoclave at 121 °C for 20 min, freeze-dried, and stored at −20 °C until further analyses. The fermented IWG samples were dispersed in 95% ethanol (1:10, *w*/*v*) and incubated at 25 °C for 24 h. The solution was then centrifuged at 16,060× *g* on a Heraeus Biofuge Pico and filtered through a 0.45 μm membrane filter. This extraction process was repeated twice, and the supernatants obtained were combined. The extract was dried under nitrogen gas and dissolved in 2 mL of 50% ethanol for further analyses.

### 2.3. Sugar Content Analysis

The sugar content was analyzed following the methods described by Murai et al. (2024) using a Dionex 5000+ high-performance anion exchange chromatographic system coupled with pulsed-amperometric detection (HPAEC-PAD) (Thermo Fisher Scientific Inc., Waltham, MA, USA) [20]. The samples were eluted isocratically with a 90 mM NaOH solution through the CarboPac PA10 anion-exchange resin column (250 × 4 mm) and its corresponding guard column (50 × 4 mm). The analyzed sugars in this study include sucrose, glucose, fructose, raffinose, stachyose, and verbascose. The sugar standards for the standard curves were purchased from Sigma-Aldrich (St. Louis, MO, USA).

### 2.4. Total Starch Content Analysis

The total starch content of the samples was determined by first removing the glucose and maltodextrins with alcohol washing based on the method described in the assay protocol for total starch analysis by Megazyme (K-TSTA-100A) [21]. The freeze-dried samples were milled and passed through a 0.5 mm screen. Approximately 5 mL of aqueous ethanol (80% *v*/*v*) was added to 100 mg of the sample and incubated between 80 and 85 °C for 5 min. The sample was vortexed, and an additional 5 mL of ethanol solution was added. The samples were then centrifuged at 3000× *g* for 10 min at 5 °C, and the supernatant was discarded. The remaining pellet was redissolved in 10 mL of additional ethanol solution and centrifuged under the same conditions, and the supernatant was discarded. The remaining procedure followed the method described by AOAC official method 996.11 [22].

### 2.5. Protein Content Analysis

The protein content was measured by the Dumas method [23], with a nitrogen conversion factor of 5.70.

### 2.6. Amino Acid Analysis

The total amino acid contents were determined following the protocols in Section 8.2 of the Product Manual for AAA-Direct, Dionex Amino Acid Analyzer (Thermo Fisher Scientific Inc., Waltham, MA, USA), with slight modifications [24]. Approximately 2–6 mg of the flour samples was hydrolyzed for 16 h at 110 °C under vacuum. The samples were then neutralized with 6 M NaOH. The column used for the separation of the amino acids was the AminoPac™ PA10 column (2 × 250 mm), along with its corresponding guard column (2 × 50 mm). The eluents, eluent gradient program, and conditions followed the specifications in Application note 163, titled “Determination of Protein Concentrations Using AAA-Direct™” (Thermo Fisher Scientific Inc., Waltham, MA, USA). External standard curves were made using the AAS18 amino acid standard mix (Millipore Sigma, Burlington, MA, USA). The result from this method includes lysine, arginine, threonine, alanine, valine, glycine, proline, serine, isoleucine, leucine, histidine, methionine, phenylalanine, aspartate, glutamate, tyrosine, and cysteine.

### 2.7. Total Phenolic Content Analysis

The total phenolic content (TPC) was determined following the method of Ritthibut et al. (2021), with some modifications [25]. The IWG extracts (100 μL) were mixed with 10% (*v*/*v*) of Folin–Ciocalteu reagent (200 μL) and 700 mM of Na_2_CO_3_ (800 μL). The mixture was then incubated at 25 °C for 2 h, and the absorbance was measured at 765 nm using a spectrophotometer. The TPC was calculated based on the standard curve with gallic acid (Sigma-Aldrich, St. Louis, MO, USA) and expressed as milligrams of gallic acid equivalent per gram of flour.

### 2.8. DPPH Radical Scavenging Activity Analysis

The 2,2-diphenyl-1-picrylhydrazyl (DPPH) radical scavenging activity was determined following the method of Ritthibut et al. (2021), with some modifications [25]. A total of 500 μL of the sample extract was mixed with 450 μL of 100 mM Tris-HCl buffer (pH 7.4) and 1 mL of 0.1 mM DPPH/methanol solution. The mixture was left at 25 °C for 30 min in the dark, and the absorbance was measured at 517 nm. The percentage of the DPPH• scavenging activity was calculated using the following equation:

(1) $$\%\;activity=\frac{Absorbance\;\left(blank\right)-Absorbance\;(sample)}{Absorbance\;(blank)}\times 100$$

### 2.9. Total Flavonoid Content Analysis

The total flavonoid content (TFC) was determined following the method of Do et al. (2014), with some modifications [26]. A total of 0.6 mL of the extract was incubated with 0.6 mL of aluminum chloride at 25 °C for 30 min, and the absorbance was measured at 415 nm. A standard curve was built with quercetin, and the TFC is reported as milligrams of quercetin acid equivalent per gram of IWG flour on a dry basis (mg/g flour).

### 2.10. Kojic Acid Content Analysis

The kojic acid content was determined following the methods by Terabayashi et al. (2010) [27]. The extracts were mixed with freshly prepared FeCl_3_ solution (1%), and the absorbance was measured at 500 nm. The kojic acid content was calculated using a standard curve built with kojic acid (Sigma-Aldrich, St. Louis, MO, USA) concentration and absorbance.

### 2.11. Statistical Analysis

The sample preparation and analysis were conducted in duplicates for a total of a quadruplicate measurement for each data point. The measurements are reported on a dry basis. ANOVA, data visualization, and Pearson’s correlation analysis were performed using Python 3.10.12 (Python Software Foundation, Wilmington, DE, USA).

## 3. Results and Discussion

### 3.1. Sugar Content

**Figure 1:**
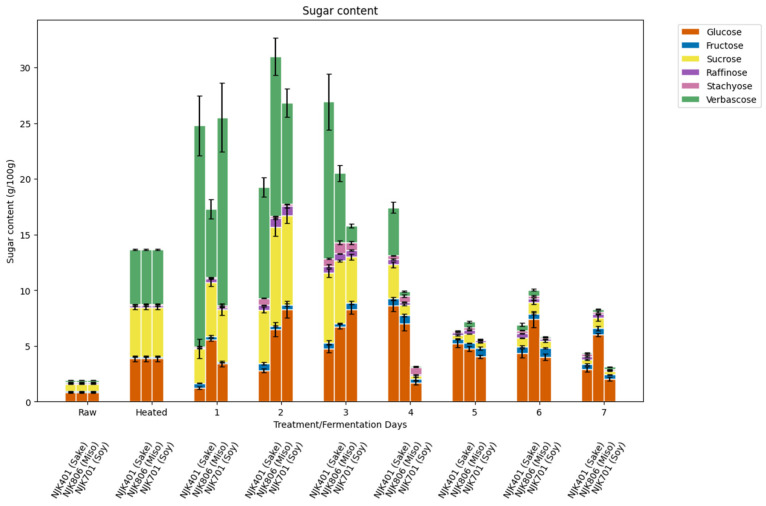
Sugar content of fermented IWG.

The most abundant sugar in the raw intermediate wheatgrass flour was glucose (0.82 g/100 g), followed by sucrose (0.65 g/100 g) (Figure 1). While Johnsrude and others (2023) [28] reported the glucose and sucrose contents of hydrolyzed feedstock-grade intermediate wheatgrass, to the best of our knowledge, the glucose and fructose contents of food-grade intermediate wheatgrass have not yet been documented. Amounts of oligosaccharides, including raffinose (0.09 g/100 g), stachyose (0.10 g/100 g), and verbascose (0.15 g/100 g), in raw intermediate wheatgrass have also never been reported to the best of our knowledge as well.

The heat treatment increased the contents of all types of sugars, where verbascose became the most abundant sugar (4.91 g/100 g), followed by sucrose (4.42 g/100 g) and glucose (3.87 g/100 g) (Figure 1). The significant increase in glucose, verbascose, and fructose following the steam treatment could be attributed to the breakdown of plant structures, such as glycoproteins. Sugars bound in glycoprotein form would not be detected by the HPAEC-PAD methodology employed in this study. Lectins, a subgroup of glycoproteins, are ubiquitously found in plants, where they serve to protect against external pathogens [29]. Research by Peumans and Van Damme (1998) highlighted that cereal grains, including wheat, rye, barley, and rice, contain lectin concentrations up to 0.5/100 g [30], suggesting that intermediate wheatgrass in its raw form is also likely to contain lectins. Additionally, research by Mattucci et al. (2004) [31] has shown that the activity of wheat germ agglutinin (WGA), a type of lectin found in wheat, decreases when heated beyond 65 °C. This behavior might help explain the increased detection of glucose, verbascose, and fructose, given that lectins have affinities for specific sugars, including glucose and galactose, which are present at the terminal ends of these compounds [32,33].

The fermentation caused a general increase in sugar content for the first three days, followed by a decrease over the subsequent days (Figure 1). Notably, the verbascose content reached 19.83 g/100 g on the first day of fermentation with the sake strain (Figure 1). Verbascose is typically found in high concentrations in peas and mung beans but not in wheat [34]. The increase in glucose could mainly be attributed to amylase enzymes produced by *A. oryzae*, which has been widely studied in the past [35]. The increase in sucrose and verbascose could suggest the presence of glycosyltransferases, which are enzymes that catalyze the biosynthesis of oligosaccharides [36]. Research by Perna et al. (2018) has shown the capability of *A. oryzae* to produce fructosyltransferase, which may explain the increase in sucrose content [37]. *A. oryzae* is also known to produce α-galactosidase [38]. While α-galactosidase is primarily known for its hydrolytic capabilities, it has also shown transglycosylation activity in certain cases [39]. β-Galactosidase has also been found to catalyze transglycosylation under certain conditions [40]. The fermentation process could have also contributed to a reduction in lectin levels, as demonstrated by the decrease in wheat germ agglutinin (WGA) activity during sourdough fermentation, highlighted in the study by Tover and Ganzel (2021) [41]. The breakdown of lectins during fermentation could have had a similar effect as the steam treatment, increasing the detectable amounts of sugars. The decrease in verbascose and sucrose concentrations could be attributed to the α-galactosidase and β-fructofuranosidase, as certain *A. oryzae* strains have been found to produce β-fructofuranosidase, which are responsible for the breakdown of sucrose molecules [42].

**Figure 2:**
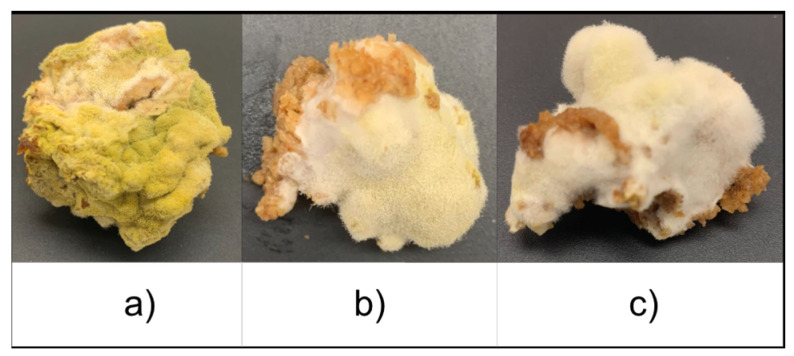
Appearance of fermented intermediate wheatgrass (IWG) with *A. oryzae* strains on day 4 of fermentation. (**a**) NJK701 (soy sauce koji); (**b**) NJK401 (sake koji); (**c**) NJK806 (miso koji).

Strains of *A. oryzae* are utilized differently in industrial applications based on their enzymatic activity. Strains with higher amylase activity are typically used for sake and miso production, while strains with higher protease activity are preferred for soy sauce production [43]. This distinction aligns with the focus of each product. In sake production, the goal is to break down starch into fermentable sugars to support alcohol fermentation, whereas in miso, sweetness plays a significant role in the overall flavor. Conversely, soy sauce production focuses on developing umami, achieved through the breakdown of proteins into amino acids by proteases. Ito and Do (1994) describe the appearance of these strains, noting that strains with strong amylase activity typically form colonies that are yellow-green to pale yellow, while those with strong protease activity are green to dark green [43]. Figure 2 shows the appearance of fermented IWG with each strain on day 4 of fermentation, which is consistent with these descriptions. The NJK401 strain (sake koji) and NJK806 strain (miso koji) both displayed a pale yellow color, whereas the NJK701 strain (soy sauce koji) exhibited a green color. However, the changes in the sugar content during fermentation did not always align with expected amylase activity levels. In some cases, the NJK701 strain produced higher sugar levels than the other strains at certain points in the fermentation period.

### 3.2. Total Starch Analysis

**Figure 3:**
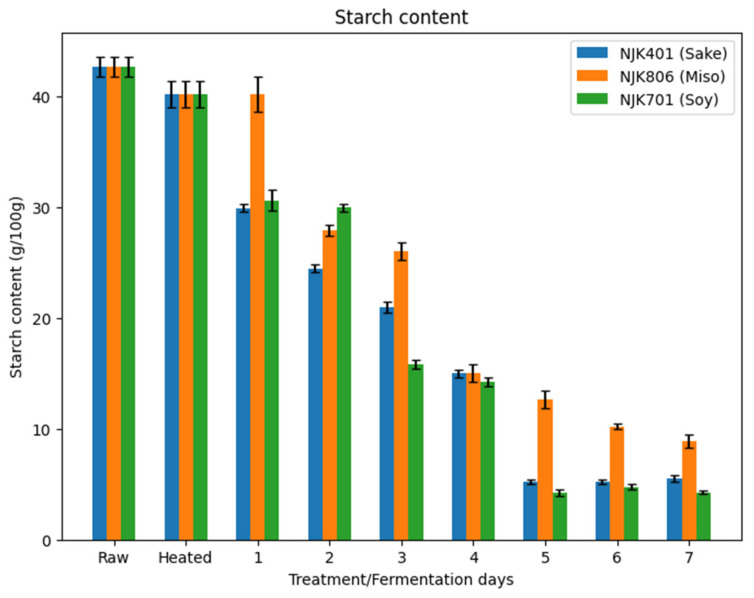
Total starch content of fermented IWG.

The starch content of the whole intermediate wheatgrass flour was 42.7 g/100 g of flour. This falls in the range (46.7–51.2 g/100 g) of starch content, as reported in past studies [44]. Heat treatment reduced the starch content (Figure 3), which corresponds to an increase in glucose content (Figure 1), consistent with previous studies showing that the thermal hydrolysis of starch leads to sugar production [45]. Fermentation continued to a decrease in the starch content, where the starch content ranged from 4.3 to 8.9 g/100 g by the seventh day of fermentation. As discussed in Section 3.1, the decrease in starch content during the fermentation could be attributed to the hydrolytic amylase enzymes produced by the *A. oryzae* strains, as widely studied in the past [37].

### 3.3. Protein Content

**Figure 4:**
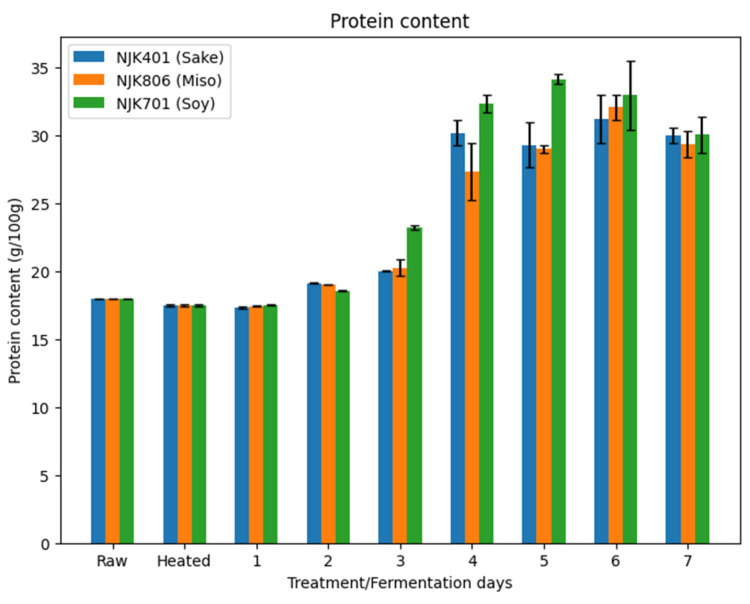
Protein content of fermented IWG.

The protein content of the whole intermediate wheatgrass flour was 18.0 g/100 g of flour (Figure 4). This is on the lower end of the reported range of protein contents of intermediate wheatgrass from past research (17.6~23.5 g/100 g) [6,44]. Fermentation significantly (*p* < 0.05) increased the protein content for all three strains, with the largest increase occurring between day 3 and 4. On day 4, there was a 50.89%, 34.88%, and 39.39% increase in protein content from day 3 for the sake, miso, and soy strains, respectively. The highest protein content was observed on day 5 (34.16 g/100 g) for the soy strain, while the highest protein content for the sake (31.21 g/100 g) and miso (32.08 g/100 g) strains was observed on day 6. A decrease in protein content was observed after the peak for all three strains. The soy strain consistently had a higher protein content than the sake and miso strains throughout the fermentation (Figure 4).

Increases in protein content for substrates fermented by *A. oryzae* have been reported in the past. For example, research by Hong et al. (2004) has shown a 10% increase in protein content in soybeans and soybean meals fermented with *A. oryzae* in a bed-packed solid fermenter [12]. Additionally, research by Rousta et al. (2021) has shown an increase in protein content in oat flour from 11% to 37% in a submerged fermentation with *A. oryzae* [46]. Such an increase in protein content can be attributed to a decrease in carbohydrate content of the fermented substrate rather than an increase in nitrogen content. As seen in Figure 1, the sugar content of the fermented substrates reaches its peak around day 2 or 3 and, depending on the strain, starts to decrease in the subsequent days. In contrast, the protein content increases during the same period the sugar content starts to decrease (Figure 1 and Figure 4). *A. oryzae*, just like many other microorganisms, utilizes glucose as its main carbon and energy source. *A. oryzae* continuously produces carbon dioxide gas throughout the fermentation process as the protease activity increases [47]. This suggests a decrease in carbon concentration of the fermented material, inversely increasing the nitrogen content and leading to an increase in the crude protein content, measured by the Dumas method through the concentration effect. A similar decrease in carbohydrate content, accompanied by an increased protein content in the fermentation substrate, has been observed in past studies [48].

### 3.4. Amino Acid Composition

**Figure 5:**
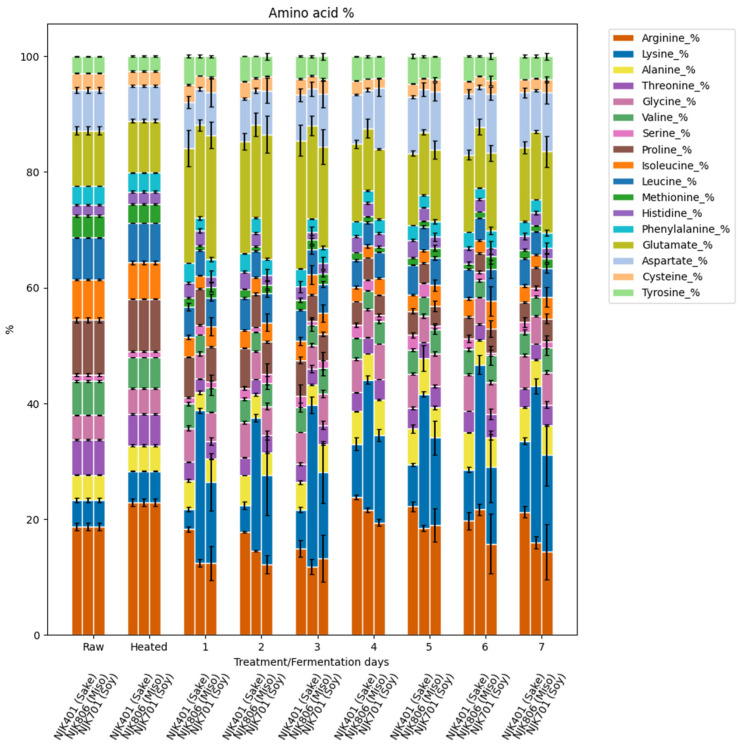
Amino acid percentage of fermented IWG.

**Figure 6:**
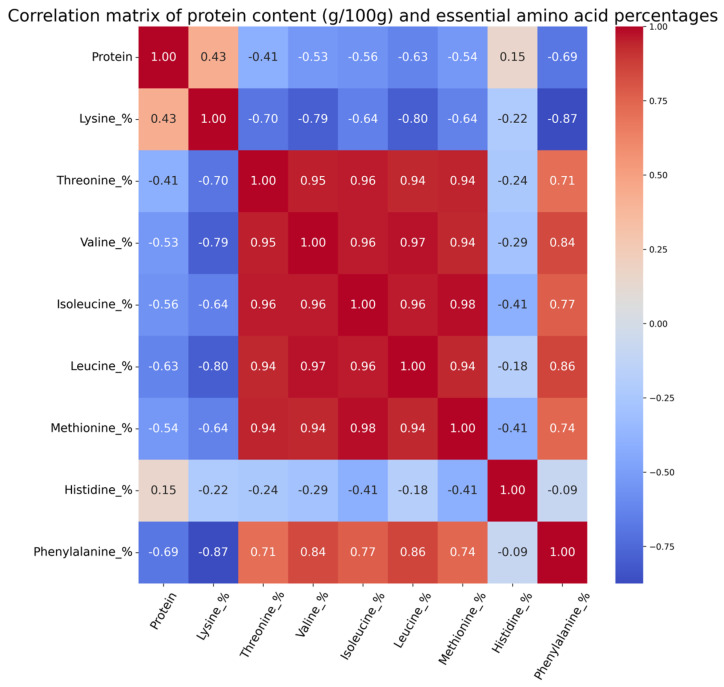
Pearson’s correlation between protein content and essential amino acids of fermented IWG.

The change in amino acid compositions of the samples through the heat treatment and fermentation are presented in Figure 5. The most abundant essential amino acids in the raw IWG samples were leucine, isoleucine, threonine, valine, and lysine. The lowest essential amino acids were histidine, phenylalanine, and methionine. While lysine accounted for 4.56% of the total amino acids in the raw IWG samples, its proportion increased progressively during fermentation. The lysine percentage reached 12.3%, 16.7%, and 27.0% in the sake, soy, and miso strains, respectively, by the seventh day of fermentation. Lysine and histidine were the only essential amino acids that had a positive correlation with the increase in protein content (Figure 6). All the other essential amino acids had a negative correlation with the increase in protein content. A similar rise in lysine content has been reported in previous studies, where the fermentation of oat flour with *A. oryzae* increased the lysine percentage from 4.35% to 7.44% of the total amino acids, excluding tryptophan [46]. The increase in lysine concentration in fermented IWG can be highly beneficial, as the current supply of plant-based lysine predominantly comes from soybean production [49]. Fermented IWG offers a promising alternative source of lysine, potentially reducing dependence on traditional crops and contributing to a more sustainable and diversified global supply of plant-based lysine.

Lysine production is a well-documented aspect of fungal metabolism, facilitated by M1 aminopeptidases encoded by the apsA and apsB genes, which liberate N-terminal lysine from peptides. However, in this study, the observed increase in lysine during fermentation cannot be fully attributed to these enzymes, as HCl hydrolysis of the flour should have liberated lysine in the unfermented samples as well. One reason for the increased proportion of lysine could be the de novo synthesis of this amino acid through the fungal α-aminoadipate pathway [50]. The pathway begins with the condensation of acetyl-CoA and 2-oxoglutarate, forming homocitrate. This intermediate then dehydrates to homoaconitate, which is then rehydrated to produce homoisocitrate. The oxidative decarboxylation of homoisocitrate generates α-ketoadipate, which is subsequently converted to α-aminoadipate through an amino transfer from glutamate. The final steps in lysine synthesis involve the sequential formation of α-aminoadipate semialdehyde, saccharopine, and, ultimately, lysine. Another reason for the change in the amino acid profile may be the ability of *A. oryzae* to assimilate ammonium through specific ammonium transporters, where ammonium plays a role in nitrogen metabolism and supports growth and development in filamentous fungi. Genomic analyses conducted by Chutrakul et al. (2024) have identified four ammonium transporter genes (*aoamt1-aoamt4*) in *A. oryzae*, with aoamt2 and aoamt3 showing ammonium-dependent expression, especially under ammonium-limited conditions [51]. The same study revealed that aoamt3 is essential for ammonium uptake, nitrogen assimilation, and filamentous growth. Overexpression of aoamt3 enhanced growth performance, increased biomass yield, and reduced glucose consumption. These findings suggest that *A. oryzae* can incorporate non-protein nitrogen, such as ammonium, into its metabolic pathways, potentially influencing the amino acid composition of the fermented products.

### 3.5. Total Phenolic Content

**Figure 7:**
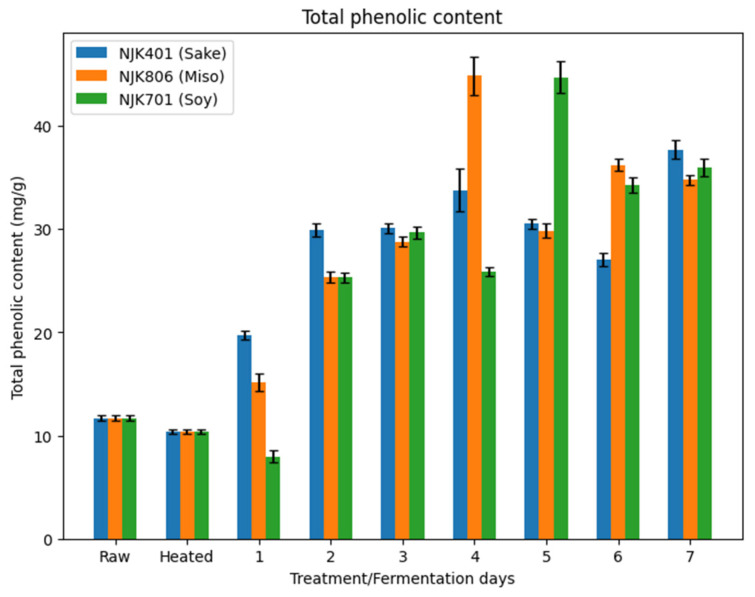
Total phenolic content of fermented IWG.

The total phenolic content of the raw IWG (11.7 mg/g) decreased following heat treatment (10.4 mg/g) (Figure 7). Combined observations from this study and past research suggest that changes in the phenolic content following a heat treatment are dependent on the conditions and substrates. For example, a study by Ghani et al. (2023) has shown that autoclaving and pasteurization of fermented broken rice do not affect TPC, while oven drying does [52]. However, a study by Musilova et al. (2020) has shown that TPC generally increases in sweet potatoes by boiling, steaming, microwaving, and baking [53]. The observed decrease in autoclaved IWG would be limited to the conditions employed in this study.

Fermentation increased the TPC for all three strains of *A. oryzae* (Figure 6). The highest TPC for the sake strain (37.7 mg/g) was observed on day 7, on day 4 for the miso strain (44.9 mg/g), and on day 5 for the soy strain (44.7 mg/g). The TPC continued to increase for days 1–3 for all strains but fluctuated in value for the subsequent days. This observation is consistent with past research by Punia et al. (2020), where the TPC of rice bran fermented with Aspergillus strains increased in the first 4 days but decreased in the subsequent 3 days [10]. As Punia et al. (2020) suggest [10], this could be due to the initial hydrolytic reactions by the enzymes produced during the SSF, releasing the bound phenolics and the degradation of gallic acid to aliphatic compounds in the later phase [54,55,56].

### 3.6. Total Flavonoid Content

**Figure 8:**
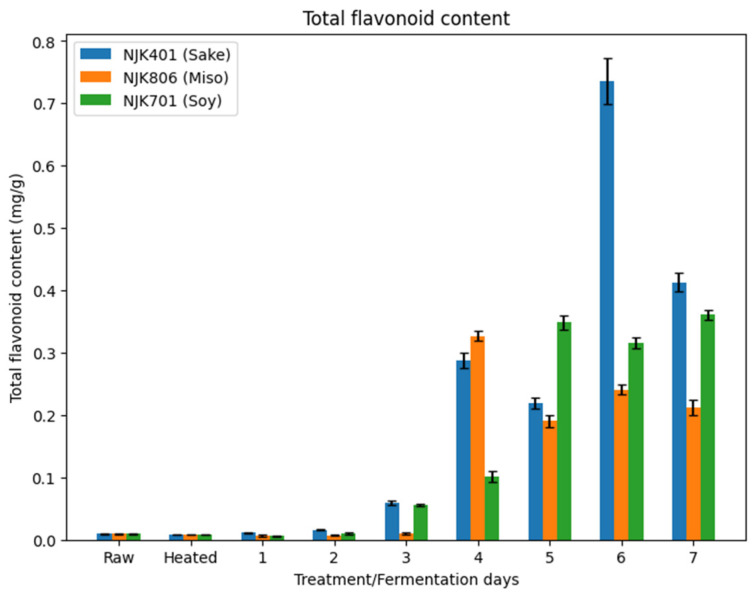
Total flavonoid content of fermented IWG.

A low flavonoid content was observed in both raw (0.01 mg/g) and heated IWG (0.01 mg/g) (Figure 8). The fermentation increased the TFC, where the sake strain (0.74 mg/g) had the highest value on day 6, the miso strain (0.33 mg/g) on day 4, and the soy strain (0.36 mg/g) on day 7 (Figure 8). As seen in the TPC, TFC also fluctuated in the later days of the fermentation. Past studies have reported mixed results on the changes in TFC during fermentation by Aspergillus strains. A study by Raffiquzzaman et al. (2015) on Japanese kelp fermented with *A. oryzae* has shown a decrease in flavonoid content over 7 days of fermentation [57]. However, a study by Cai et al. (2011) has shown an increase in TFC for oats fermented with Aspergillus strains for 3 days [58]. An increased flavonoid content in the fermented IWG could enhance its functional properties, offering potential health benefits, such as antioxidant, anti-inflammatory, and neuroprotective effects, making it a valuable ingredient for both food and nutraceutical applications [59].

Flavonoids are secondary metabolites found ubiquitously in plants, as they regulate cell growth and response to biotic and abiotic stresses [60]. As with the increase in TPC, the increase in TFC could be due to the release of bound flavonoid compounds, increasing their detectable amount [55]. Free phenolic compounds, including free flavonoids, are found in the vacuoles of plant cells, whereas bound flavonoids are attached to the cell wall structures. As Huynh et al. (2014) discuss, increased flavonoid content could be due to the enzymes produced by the *A. oryzae* strains, such as cellulase, glucosidase, and pectinase [55].

### 3.7. DPPH Radical Scavenging Activity

**Figure 9:**
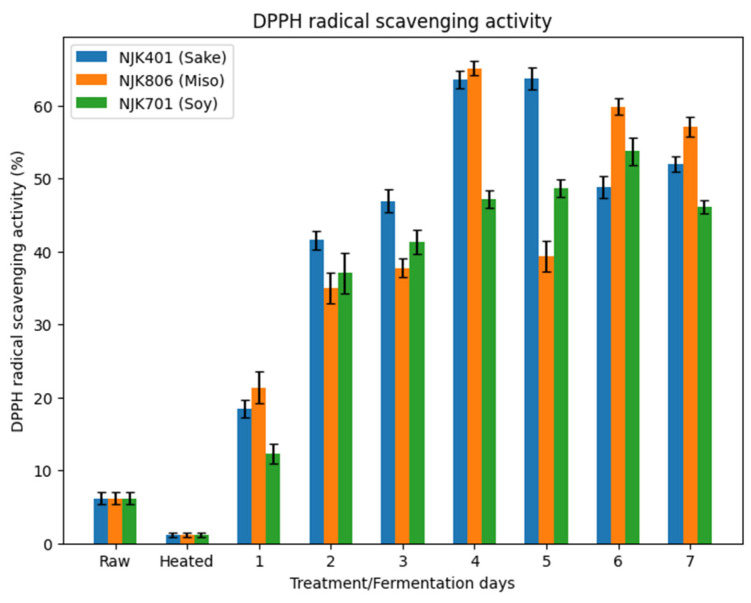
DPPH radical scavenging activity of fermented IWG.

**Figure 10:**
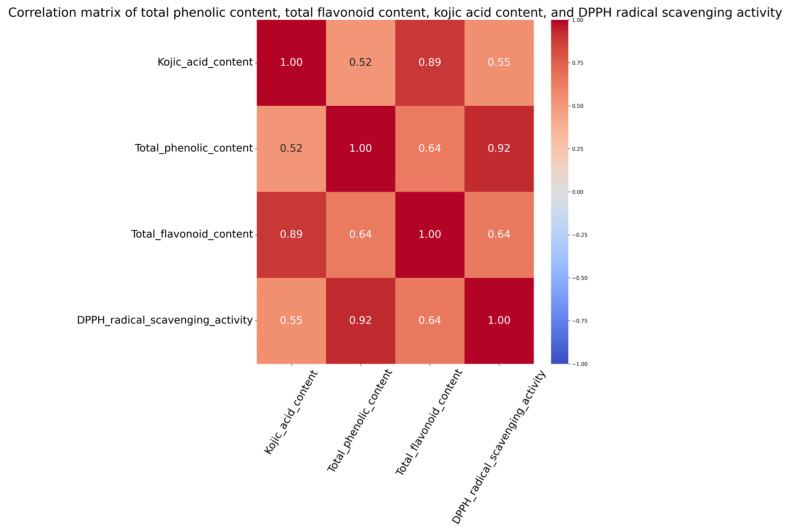
Pearson’s correlation between kojic acid content, total phenolic content, total flavonoid content, and DPPH radical scavenging activity of fermented IWG.

The DPPH radical scavenging activity in the heat-treated samples (1%) decreased from the raw samples (6%), while the fermentation process increased the scavenging activities for all three strains of *A. oryzae* (Figure 9). The decrease in the scavenging activity following the steam treatment is contrary to some research, where the scavenging activity has been found to increase in several cases [61,62]. The decrease in the scavenging activity may be due to the decreased TPC following the heat treatment. Past research has shown TPC and the DPPH radical scavenging activity to be positively related [25,63], which is similar to what is observed in this study (r = 0.92) (Figure 10).

The highest activity for the sake strain (64%) was observed on the fifth day, on the fourth day for the miso strain (65%), and on the sixth day for the soy strain (53%). This result is similar to research conducted by Rhittibut et al. (2021), where fermentation with Aspergillus strains increased the scavenging activity of rice bran above 60% [25]. The DPPH radical scavenging activity stabilized from day 4 and beyond, similarly to the TPC (Figure 7). An increased DPPH radical scavenging activity may enhance the quality of IWG by preventing fat rancidity caused by oxidation, thereby improving its shelf life and stability [64]. Additionally, high antioxidant activity can protect against oxidative stress in organisms, reducing the risk of degenerative diseases, such as cardiovascular disease, Alzheimer’s, and cancer, further positioning fermented IWG as a functional food with broad applications [65].

### 3.8. Kojic Acid Content

**Figure 11:**
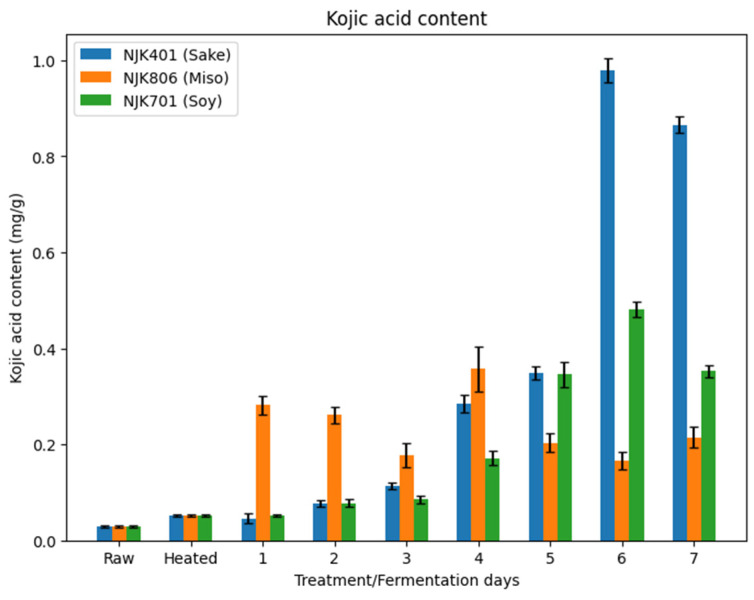
Kojic acid content of fermented IWG.

While the raw IWG (0.03 mg/g) and heat-treated IWG (0.05 mg/g) contained little kojic acid, all three strains of *A. oryzae* significantly increased the kojic acid content during the fermentation process (Figure 11). The sake (0.98 mg/g) and soy strain (0.48 mg/g) achieved the highest kojic acid content on day 6, and the miso strain achieved the highest kojic acid content on day 4 (0.36 mg/g). Kojic acid was found to be positively correlated (r = 0.89) with the total flavonoid content (Figure 10). While kojic acid is not a flavonoid, this result may suggest an interconnected fungal biosynthetic mechanism of kojic acid and flavonoid production. The increase in kojic acid is expected, since it is a product of fungal secondary metabolism produced mainly by *Aspergillus* species in the current industry [17]. Kojic acid is known to have multiple applications, including, but not limited to, as a whitening agent and in antibacterial and antioxidant products [66,67,68]. As summarized by Felipe et al. (2023), the fermentation temperature for kojic acid production typically ranges between 25 and 30 °C, but other factors, such as carbon source, nitrogen source, pH, and cultivation, can vary [17]. Typical carbon sources include glucose and sucrose, which may explain some of the loss of glucose and sucrose and the increase in kojic acid content seen within this study (Figure 1). These results highlight the potential of IWG as a substrate not only for food and animal feed applications but also for high-value products, like kojic acid for cosmetic and pharmaceutical uses. Further studies are needed to optimize kojic acid production using IWG as a substrate, considering the effects of substrate composition and fermentation conditions.

## 4. Limitations

The results of this study are limited to the lot of intermediate wheatgrass obtained from the Perennial Pantry at the time of the experiment, the strains of *A. oryzae*, and the fermentation conditions employed in this study.

## 5. Conclusions

SSF with *A. oryzae* has historically been used to enhance the nutritional value of cereal grains and is gaining increased attention for industrial applications. In this study, we demonstrated the potential of applying SSF to IWG, a novel perennial crop with significant ecological benefits. The fermentation increased the protein, lysine, total phenolic, total flavonoid, and kojic acid content of IWG. The observed increase in total phenolic and total flavonoid content, along with the production of kojic acid, highlights the potential of fermented IWG as a functional food ingredient with antioxidant, anti-inflammatory, and other health-promoting properties. Sugar content initially increased during the first two to three days of fermentation, followed by a decline in later stages. Analysis using HPAEC-PAD identified oligosaccharides such as raffinose, stachyose, and verbascose—compounds typically associated with legumes—in the fermented IWG. Findings from this study align with past research, showing that SSF concentrates protein content rather than increasing its net amount, as carbohydrates are metabolized and lost during fermentation. The results from this study, combined with past research on SSF with *A. oryzae* on other grains, suggest that extending fermentation beyond four days may not further enhance TPC or the DPPH radical scavenging activity. Future studies could explore the specific enzyme activities of each strain and their interactions with IWG as a substrate. Moreover, research could investigate producing traditional fermented foods, such as sake, miso, and soy sauce, using IWG, comparing their quality, flavor, and nutritional properties to products made from conventional grains. These findings underscore the potential of SSF with *A. oryzae* as an effective method to enhance the nutritional and functional properties of IWG, supporting its broader application in sustainable food and feed systems.

## Data Availability

The original contributions presented in this study are included in the article. Further inquiries can be directed to the corresponding author.
